# Integrative characterization of germ cell-specific genes from mouse spermatocyte UniGene library

**DOI:** 10.1186/1471-2164-8-256

**Published:** 2007-07-28

**Authors:** Eunyoung Choi, Jiae Lee, Jungsu Oh, Inju Park, Cecil Han, Chongil Yi, Do Han Kim, Byung-Nam Cho, Edward M Eddy, Chunghee Cho

**Affiliations:** 1 Department of Life Science and Research Center for BiomolecularNanotechnology, Gwangju Institute of Science and Technology, Gwangju 500-712, Korea; 2 Department of Life Science, The Catholic University of Korea, Buchon 421-743, Korea; 3 Gamete Biology Section, Laboratory of Reproductive and Developmental Toxicology, National Institute of Environmental Health Sciences, National Institutes of Health, Research Triangle Park, North Carolina 27709, USA

## Abstract

**Background:**

The primary regulator of spermatogenesis, a highly ordered and tightly regulated developmental process, is an intrinsic genetic program involving male germ cell-specific genes.

**Results:**

We analyzed the mouse spermatocyte UniGene library containing 2155 gene-oriented transcript clusters. We predict that 11% of these genes are testis-specific and systematically identified 24 authentic genes specifically and abundantly expressed in the testis via in *silico *and *in vitro *approaches. Northern blot analysis disclosed various transcript characteristics, such as expression level, size and the presence of isoform. Expression analysis revealed developmentally regulated and stage-specific expression patterns in all of the genes. We further analyzed the genes at the protein and cellular levels. Transfection assays performed using GC-2 cells provided information on the cellular characteristics of the gene products. In addition, antibodies were generated against proteins encoded by some of the genes to facilitate their identification and characterization in spermatogenic cells and sperm. Our data suggest that a number of the gene products are implicated in transcriptional regulation, nuclear integrity, sperm structure and motility, and fertilization. In particular, we found for the first time that Mm.333010, predicted to contain a trypsin-like serine protease domain, is a sperm acrosomal protein.

**Conclusion:**

We identify 24 authentic genes with spermatogenic cell-specific expression, and provide comprehensive information about the genes. Our findings establish a new basis for future investigation into molecular mechanisms underlying male reproduction.

## Background

During male reproduction, germ cells are processed from the primordial stage through spermatogenesis occurring in the seminiferous tubules of testis. The tightly regulated process that occurs in mitotic, meiotic, and post-meiotic phases drives successful male germ cell development or spermatogenesis [1-3]. Spermatogonial stem cells located around the outer region next to the basal lamina surrounding seminiferous tubules undergo mitosis, and some differentiate into later-stage spermatogonia that gradually become primary spermatocytes. At this stage, the most important event, meiosis, is additionally required. These cells continue through the first meiotic division to become secondary spermatocytes. A second meiotic division occurs in rapid succession to produce haploid spermatids that are remodeled into spermatozoa by spermiogenesis. The tight modulation of this developmental process suggests the expression of a highly organized network of genes. The regulation of gene expression during spermatogenesis occurs at three levels, namely the intrinsic, interactive, and extrinsic levels [3]. The intrinsic program determines which genes are utilized and when genes are expressed in germ cells. The interactive process between germ cells and somatic cells is necessary for germ cell proliferation and progression, and is regulated by extrinsic influences, such as steroid and peptide hormones. Notably, the intrinsic genetic program involves germ cell- and stage-specific gene expression.

Recently, several studies have focused on the identification of cell- and tissue-specific transcriptomes using high-throughput genomics. While earlier studies have provided inclusive information about testicular genes, the identities and characteristics of spermatogenic cell-specific genes are largely unknown [4-9]. The UniGene database contains an extensive collection of information about sets of transcript sequences, including ESTs (Expressed Sequence Tags). UniGene transcripts are organized into clusters composed of fragments of sequences overlapping with at least one other member of the same cluster. GenBank sequences are automatically partitioned into non-redundant sets of gene-oriented clusters [10]. Consequently, each cluster contains sequences corresponding to a single gene, and related information, such as gene expression patterns and mapping positions analyzed *in silico*. Thus, the UniGene database provides significant information (a combination of gene sequences and computational bioinformatics) to facilitate the prediction of gene expression and function from each cluster.

Comprehensive understanding of male germ cell development and fertilization requires discovery and functional characterization of germ cell-specific genes, because they are highly unique processes that do not occur in any other tissue. Previously, we identified and characterized a number of novel genes from the round spermatid UniGene library [11]. The spermatocyte stage is also a significant period during which meiosis occurs and haploid daughter cells are produced. Here, as an ongoing study on germ cell-specific genes, we identify 24 spermatogenic cell-specific genes, using sequence information from the spermatocyte UniGene database, and analyze their characteristics at the gene and transcript levels. Further, we characterized proteins encoded by the genes and predict that a number of them have significant functions in various processes during spermatogenesis and fertilization. Thus, we report the efficient and unique systematic identification and in-depth characterization of unexplored genes specific to spermatogenic cells.

## Results

### The spermatocyte UniGene library and *in silico* selection of gene candidates

The McCarrey Eddy spermatocyte library of mus musculus (Lib.6787), one of the largest spermatogenic cell libraries deposited in the UniGene database at NCBI [12], was analyzed to classify gene entries into known and unknown genes, or testis-specific and non-testis-specific genes. As of April 2006 (*Mus musculus *UniGene Build #156), the spermatocyte library consisted of 2155 UniGene entries. We classified the genes from the library on the basis of the following criteria: (i) Genes previously named or assigned with potential functions were classified as 'known', and genes with unassigned functions were regarded as 'unknown'. (ii) If all of ESTs of a given gene were found only in testis and/or spermatocyte, or the numbers of testis or spermatocyte ESTs in a cluster were higher (nine times or more) than that of non-testis and/or spermatocyte ESTs, the gene cluster was selected as 'testis-specific'. According to these criteria, about three-fourth of the genes were classified as known/named, and 112 were testis-specific. On the other hand, we selected 544 clusters as unknown genes, of which 118 were testis-specific (Table 1). Although three quarters of the total gene entries are named/known genes, similar numbers of testis-specific genes were found among the known and unknown candidates, indicating that many of the testis-specific genes remain to be characterized. Taken together, the combination of known and unknown testis-specific genes comprises 11% of the spermatocyte UniGene entries.

**Table 1:** Classification of genes in the spermatocyte library

Genes	Number
Total entries (as of Aril 2006)	2155
Known (named)	1611
Testis specific	112
Unknown	544
Testis specific	118
Total entries (as of March 2003)	1218
Unknown, testis specific	134
Analyzed in vitro	81
Authentic, testis specific	24

Gene entries from the spermatocyte UniGene library were classified into known and unknown genes, or testis-specific and non-testis-specific genes. Genes with >90% testicular ESTs of total ESTs, including three non-testicular tissues at most, were classified as testis-specific genes. One hundred thirty four genes selected from the earlier version of the library were analyzed *in vitro *to determine whether they are authentic genes with abundant and evident testis-specific expression. All of the 134 genes are listed in Table 2.

At the beginning of our study (March 2003), the total number of gene entries within the earlier version of the spermatocyte library was 1218. A search for testis-specific genes with unknown or unassigned functions resulted in the selection of 134 candidates and these genes were further analyzed in the present study (Tables 1 and 2).

**Table 2:** Analysis of unknown, testis-specific gene candidates

No	ID	Coding seq.	PCR		Northern	No	ID	Coding seq.	PCR		Northern
									
			E	T					E	T	
1	Mm.46140	+	+	-		68	Mm.84572	+	+	+	+
2	Mm.153895	+	-			69	Mm.373242	+	+	+	+
3	Mm.85000	+	+	-		70	Mm.84567	+	+	-	
4	Mm.67591	-				71	Mm.81022	+	+	+	-
5	Mm.425335	+	+	+	+	72	Mm.73639	-			
6	Mm.45833	+	+	+	+	73	Mm.290718	+	+	+	+
7	Mm.26987	+	+	-		74	Mm.66965	+	+	-	
8	Mm.232593	+	+	+	-	75	Mm.55870	+	+	+	+
9	Mm.157041	-				76	Mm.50068	+	+	+	-
10	Mm.152558	+	-			77	Mm.46472	-			
11	Mm.152543	+	-			78	Mm.46159	+	+	+	+
12	Mm.138549	+	+	-		79	Mm.437189	+	+	+	+
13	Mm.107775	+	+	+	+	80	Mm.116803	+	+	+	+
14	Mm.86807	+	+	+	-	81	Mm.46144	+	-		
15	Mm.86671	+	+	+	+	82	Mm.45824	+	+	+	+
16	Mm.63791	-				83	Mm.45377	+	-		
17	Mm.5349	+	-			84	Mm.33810	-			
18	Mm.157509	+	-			85	Mm.329058	+	+	+	+
19	Mm.116959	+	+	-		86	Mm.257647	-			
20	Mm.87381	+	+	-		87	Mm.257295	-			
21	Mm.87368	+	-			88	Mm.250945	+	+	+	-
22	Mm.85045	+	+	+	+	89	Mm.249832	+	+	-	
23	Mm.333010	+	+	+	+	90	Mm.23578	-			
24	Mm.263708	+	+	+	+	91	Mm.234186	-			
25	Mm.72810	+	+	+	-	92	Mm.23377	+	+	+	+
26	Mm.67234	+	+	+	+	93	Mm.23356	+	+	+	-
27	Mm.660	+	+	-		94	Mm.233179	-			
28	Mm.63782	+	+	-		95	Mm.230316	-			
29	Mm.61136	-				96	Mm.228181	-			
30	Mm.60992	+	+	-		97	Mm.216973	-			
31	Mm.56177	+	+	+	-	98	Mm.210318	-			
32	Mm.52285	+	-			99	Mm.210312	+	+	+	-
33	Mm.46124	+	+	-		100	Mm.195791	+	-		
34	Mm.45462	+	+	-		101	Mm.271248	-			
35	Mm.33629	+	+	+	-	102	Mm.165697	+	+	+	+
36	Mm.30958	+	+	+	-	103	Mm.191144	-			
37	Mm.257958	+	+	-	-	104	Mm.188575	+	+	+	-
38	Mm.250904	-				105	Mm.176944	-			
39	Mm.250670	-				106	Mm.175502	-			
40	Mm.238261	+	-			107	Mm.172330	-			
41	Mm.23534	+	+	+	+	108	Mm.172329	-			
42	Mm.210311	+	-			109	Mm.172326	-			
43	Mm.20109	-				110	Mm.172324	-			
44	Mm.192428	+	+	-		111	Mm.171540	-			
45	Mm.18549	+	-			112	Mm.159795	+	+	+	+
46	Mm.171269	+	-			113	Mm.159590	+	+	-	
47	Mm.170672	-				114	Mm.158502	+	-	-	
48	Mm.159587	-				115	Mm.158267	+	+	+	-
49	Mm.157881	-				116	Mm.158164	+	-		
50	Mm.157632	+	-			117	Mm.157767	+	+	+	+
51	Mm.153685	+	-			118	Mm.157538	-			
52	Mm.152610	-				119	Mm.157531	-			
53	Mm.152605	+	+	-		120	Mm.157510	-			
54	Mm.152599	+	+	-		121	Mm.157006	-			
55	Mm.152597	+	-			122	Mm.152611	-			
56	Mm.152594	-				123	Mm.152606	-			
57	Mm.159281	+	+	+	+	124	Mm.152604	-			
58	Mm.152587	+	-			125	Mm.152601	-			
59	Mm.152586	-				126	Mm.152600	+	-		
60	Mm.152544	-				127	Mm.152598	+	-		
61	Mm.252733	+	+	+	+	128	Mm.152541	-			
62	Mm.148821	+	-			129	Mm.139088	-			
63	Mm.128120	-				130	Mm.132427	-			
64	Mm.11014	-				131	Mm.109314	+	-		
65	Mm.109877	-				132	Mm.108732	-			
66	Mm.101915	+	-			133	Mm.103240	-			
67	Mm.99172	-				134	Mm.101717	-			

Gene candidates were subjected to *in silico *and *in vitro *analyses. Genes with potential coding sequences were analyzed by RT-PCR in which those expressed (E) as correctly-sized PCR products were further analyzed to determine whether they are testis-specific (T). Lastly, to investigate transcript size and expression level, genes with testis-specific expression were subjected to Northern blot analysis. Genes with positive and negative results are indicated as + and -, repectively.

### Testicular expression of genes

**Figure 1:**
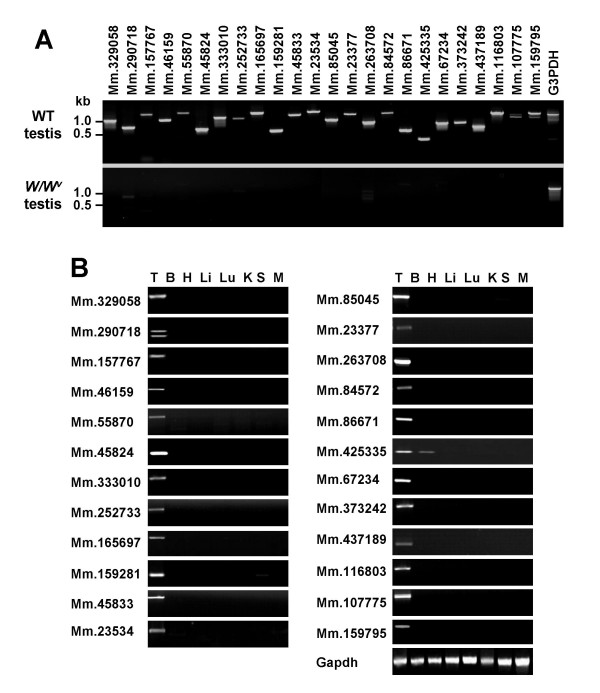
Testicular expression of the genes. **(A) **Complementary DNA from whole testes of wild-type (WT) mice and germ cell-lacking testes of W/W^v ^mutant mice were amplified by PCR. All the genes were expressed in wild-type testis but not testis deficient in germ cells, suggesting germ cell-specific expression. The faint bands observed in the germ cell-lacking testes for some of the genes are likely to be non-specific signals, since their sizes are different from those shown in WT testis. **(B) **Genes were analyzed by RT-PCR in eight adult male mice tissue types. All genes except Mm.425335 were exclusively expressed in the testis. The glyceraldehyde-3-phosphate dehydrogenase (Gapdh) band intensity was used to estimate equivalent amounts of cDNA templates among tissues. T, Testis; B, Brain; H, Heart; Li, Liver; Lu, Lung; K, Kidney; S, Spleen; M, Skeletal muscle.

**Figure 2:**
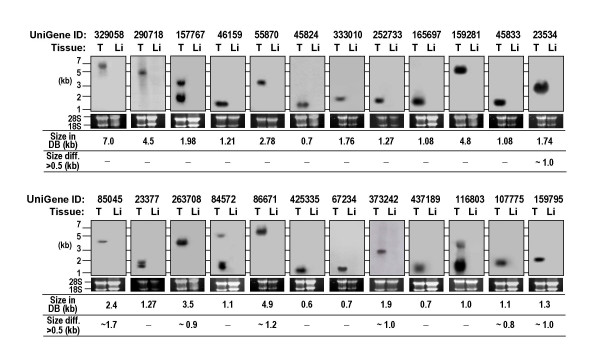
Transcript analysis of the 24 genes. Total RNA from adult testis (T) and liver (L) was hybridized with cDNA probes of the 24 genes. Agarose gels were stained with ethidium bromide to visualize 28S and 18S RNA. Seven genes display significant differences in transcript size between Northern blots and those predicted from the UniGene database (DB). Transcript sizes from known sequences (UniGene database), and transcripts with significant differences in size between the Northern blots and cDNA sequences are indicated below the blots.

To determine whether the candidates selected from the UniGene library are true genes with testis-specific expression, various analyses were performed (Table 2). In total, 134 gene candidates were analyzed with regard to whether their open reading frames contained reliable amino acid coding regions. We regarded a certain gene candidate as a gene with a reliable amino acid coding region if the size of the longest coding sequence is larger than at least 20% of that of an entire transcript (mRNA) sequence in the gene. Of these, 81 genes encoded possible amino acid sequences, while remaining 53 candidates showed that deduced coding sequences are too short, compared to transcript sizes (i.e., A deduced coding region comprises less than 20% of an entire transcript sequence), or displayed unreliable coding regions (multiple small coding regions). Accordingly, these 53 genes were eliminated from further analyses. Reverse transcription-polymerase chain reaction (RT-PCR) analysis of the 81 candidates resulted in the amplification of PCR products with the expected sizes in 56 candidates. However, we observed no or incorrectly sized PCR products of the remaining 25 candidates from testes, which were thus excluded from further analysis. Tissue distribution was investigated by PCR using mouse cDNA from eight different tissues. Out of the 56 candidate genes, 37 were identified as testis-specific or predominant (Table 2). Gene expression data from 24 of the 37 genes are shown in Figure 1, since subsequent Northern blot analysis revealed abundant expression only in these genes (see below, and Table 2 and Figure 2). All the gene transcripts were amplified with the correct sizes (Table 3 and Figure 1A), and specifically or predominantly expressed in the testis (Figure 1B). Spermatogenesis occurs in seminiferous tubules containing a mixture of germ cells and somatic cells, such as Sertoli cells. None of the genes were transcribed in germ cell-lacking testes of *W/W*^*v *^mutant mice (Figure 1A).

**Table 3:** List of genes and gene specific primers designed for RT-PCR analysis

UniGene ID	GenBank ID	PCR primers		Size (bp)
			
		Forward (5'-3')	Reverse (5'-3')	
Mm.329058^a^	AB097085	AAGGCTTCTCTGTCGGATCG	TGCACTCCAGGTTCAAACAG	797
Mm.290718^a^	DQ864732	TTGCAAGACTAGGGATGGTG	TGCCAGAGAGTGTGAGAGGG	588
Mm.157767	AK077190	ACTGTACCTGCTGTGACTGC	TTTGTGTTCAGGGTCTCCAG	979
Mm.46159^a^	AK005850	CTGCAAGTTCCTGTGTTCTG	CAGGGATGACAAGGACAACG	767
Mm.55870	AK029548	TTCTCAGGCTTCCAGGTATG	GGGAGCAAGCCAGTAAGCAG	1025
Mm.45824^a^	AK005610	GTCCCTCTGTAAGTCTGAAG	TGGACACTCTTTGGACACAG	538
Mm.333010	AK030264	GATTGCGACAGTTGACAGTG	TGATCCATTGTACAGACGGC	862
Mm.252733	AK006257	GCCGTGATCTCCAAAATGTC	ACAGCTTCAGTGCGTTCTTC	810
Mm.165697	AK006079	GGCTTTAGTGAGGACCTTGG	ACAGTCCCTAGCTCAGATTG	968
Mm.159281	XM_127913	TAACGGGAAGAAGGTGGAAG	AGGAGGGTCTGTAAGGTAGC	475
Mm.45833^a^	NM_133711	AGATTGTTTACCACTGCCGG	CTACTTCCTCCATGTCCTTC	914
Mm.23534^a^	AK016718	TTCCTCACGGCACAACTCAG	GTGTCCTCCGAATCCCTCAG	997
Mm.85045	AK019748	CCTGTATCTTCACTGCCTCG	AGTTCTCGTTTCAGCCAGGG	772
Mm.23377^a^	AF522069	CCCTAGAGCTCTGTCCTTGG	CAGACATCAGCTCGGCCAAC	997
Mm.263708	AK006048	TCTCGGGCATCCTTCTGGGC	GGCTGAGAAGGTGTGGGCG	681
Mm.84572	AK006488	CCTTTTGGGGTGCACTTGGC	AGAGAGACAACCAAGTGAGC	1014
Mm.86671	AK133200	TTAAGTCCTGCTGCTGTTGG	ATGCCTCAAGGGAACTCCAG	440
Mm.425335	AK005943	TTCTTTCTCAGGTGCCGATG	CTTTCTGTGACATGTGACGC	254
Mm.67234	AK018916	AAGATGTCCTGTGGCCCTGG	GCCAGACAGAACAGGTAGTG	639
Mm.373242	AK077102	CAGCATAGGCGCAGAAAGAG	AGTCCCAGAGAAGAGGCCAG	683
Mm.437189^a^	AF463501	CTATGACTCGGGTGGCCAAG	TGGCTTTGACAGGACCACAG	545
Mm.116803^a^	AK014971	CTCTCTCACCTCCGTTCCAG	GAGTGAGAAAGGCAAGGAAG	1009
Mm.107775	AK015444	CATCTGCTCTGGTCCTGCTG	GACAACCCCATTCTATCCCG	876
Mm.159795^a^	AK014942	TCGGGCTCACTGTTTGCTAC	TGAAGGAGAGACTAGTGCTG	964

^a ^The following genes were named previously: Mm.329058, Tudor domain containing 6 (Tdrd6) [47]; Mm290718, Zinc finger protein 541 (Zfp541); Mm.46159, Ring finger protein (Rnf151); Mm.45824, Spermatogenesis associated 19 (Spata19); Mm.45833, Spermatogenesis associated 4 (Spata4) [48]; Mm.23534, Tektin 3 (Tekt3) [23]; Mm.23377, Testis expressed gene 22 (Tep22) [22]; Mm.437189, Cysteine-rich perinuclear theca protein 4 (Cypt4) [20]; Mm116803, HRAS-like suppressor family, member 5 (Hrasls5); and Mm.159795, Cation channel, sperm associated 3 (CatSper3) [21].

### Transcript analysis of genes

To determine the expression levels and transcript sizes of the gene candidates, we performed Northern blot analysis (Figure 2). For 24 of the 37 genes, significant signals were detected in total RNA samples from testis, but not those from liver tissue (used as a negative control). These results are consistent with tissue distribution data obtained by RT-PCR. Testicular transcript sizes ranged from ~1 kb (Mm.425335) to 7 kb (Mm.329058). For 17 genes, transcript sizes determined by Northern blotting were comparable to those estimated from the UniGene database, while for the other seven genes, appreciable differences in transcript size (> +/- 0.5 kb) were evident between Northern blots and UniGene database sequences (Figure 2). Thus, the transcript sequences for the 17 genes can be regarded with confidence as full-length cDNAs or sequences containing the majority of entire cDNA sequences. The Northern blot analysis also revealed that four genes produce transcripts with more than a single size (Figure 2), suggesting the presence of multiple transcript isoforms in these genes by alternative splicing.

### Developmental expression patterns of genes

**Figure 3:**
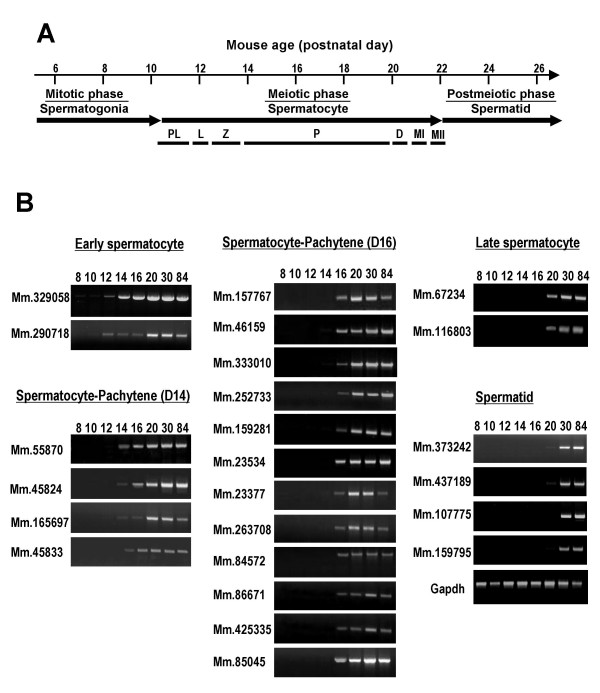
Developmental expression pattern of the genes during spermatogenesis. **(A) **Schematic diagram of juvenile spermatogenesis composed of mitotic, meiotic and postmeiotic phases. Meiotic prophase includes preleptotene (PL), leptotene (L), zygotene (Z), pachytene (P) and diplotene (D) stages. Spermatocytes at diplotene undergo rapid meiotic divisions (MI and MII). **(B) **Gene expression was analyzed by RT-PCR using cDNAs prepared from mouse testis at different days after birth. Genes were divided into five groups based on their expression timing. Gapdh, glyceraldehyde-3-phosphate dehydrogenase.

To establish the developmental expression patterns of the 24 genes during spermatogenesis, RT-PCR analysis was performed using mouse testis cDNA obtained at different time-periods after birth (8-84 days). During spermatogenesis in prepubertal mouse, primordial germ cells proliferate and differentiate increasingly to produce spermatogonia, spermatocytes, and spermatids (Figure 3A) [13]. If a particular gene is expressed in germ cells during spermatogenesis, the corresponding transcript will appear in the testis at a post-partum time-point corresponding to the specific stage of spermatogenesis.

RT-PCR data disclosed that all the genes are expressed at least after day 12, indicative of germ cell-specific and developmentally regulated expression (Figure 3B). The 24 genes could be divided into 5 clusters, based on expression patterns. The first cluster, including two genes, is expressed from the early spermatocyte (leptotene and zygotene) stage generated from 12-day-old postnatal mouse testes. The second and third clusters, the pachytene spermatocyte stage, is generated from 14- and 16-day-old postnatal mouse testes, respectively, and contain 16 genes. Two genes comprising the fourth cluster were expressed from the late spermatocyte stage generated from 20-day-old postnatal mouse testes. The fifth cluster involving the spermatid stage contained four genes. It should be noted that the majority of the genes are expressed at the spermatocyte stage from which the genes were selected *in silico*. Genes encoding ADAM2 (a disintegrin and metalloprotease 2) and protamine 2, of which expression starts during and after meiosis, respectively, were used for controls (data not shown).

### *In silico* analysis of genomic, transcript, and protein characteristics

**Figure 4:**
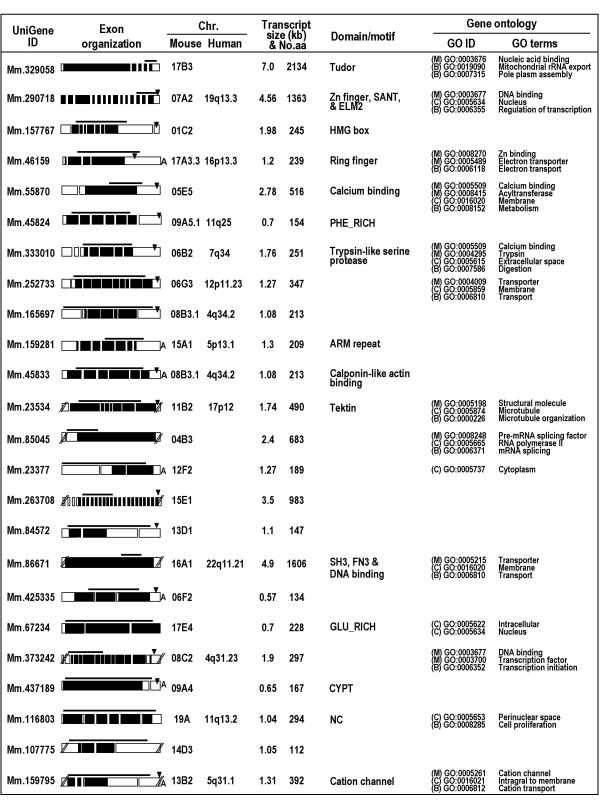
Genomic, transcript, and protein characteristics of the genes in silico. Gene structure and exon organization were determined using genome database searches. In exon organization, the boxes represent exons. The bars indicate regions amplified in the PCR analysis and used as probes in the Northern blot analysis. Coding regions were determined by selecting the longest open reading frames deduced from cDNA sequences. The predicted coding regions are shaded. The position of the poly A signal is marked by filled arrowheads, and the presence of poly A is indicated by 'A'. Chromosomal location was determined by searching the mouse and human genome databases. The predicted amino acid sequences of genes were analyzed using various bioinformatics tools (see Experimental Procedure). For annotation of genes with ontology terms, amino acid sequences were submitted to and subsequently obtained from exclusive web servers (Goblet), which use a variety of different protein databases and provide gene ontology codes. Each gene ontology code falls into one of the larger categories of molecular function (M), cellular component (C), or biological process (B).

To characterize genomic, transcript and protein natures of the genes, we performed various database searches. Figure 4 shows exon organization, chromosomal locations, transcript sizes, numbers of amino acids, specific domain/motif, and gene ontology of the predicted proteins encoded by the genes. The exon numbers in the genes are variable, ranging from 1 to 27 exons. The novel genes are widely distributed on mouse chromosomes. To extend these findings on mouse genes, we searched the human genome database for orthologs. Human orthologs for 13 mouse genes were present in genomic regions of conserved synteny between mice and humans. The other 11 genes did not have human orthologs, suggesting differential expansion in the mouse genome. The protein-coding region of each gene was defined by selecting the longest amino acid sequence terminating before a polyadenylation signal (if there is one present), and deduced amino acid sequences were subjected to database searches. Nineteen gene products were predicted to contain various domains and motifs, and found to be annotated with gene ontology codes. Thus, based on the *in silico *information, some of these proteins are predicted to be implicated in transcriptional regulation and/or nuclear activity (Mm.290718, Mm.157767, Mm.85045, Mm.86671 Mm.373242, Mm.437189 and Mm116803), metabolic processes (Mm.46159, Mm.55870, Mm.333010, Mm.252733, Mm86671 and Mm.159795) and cell structure (Mm.45833 and Mm.23534) (Table 4).

**Table 4:** Putative functions of the eight gene products in reproduction

Function	UniGene	*In silico *information	Localization in GC-2 cells	Presence in TSC	Presence in MS
Transcriptional regulation	Mm.290718	Transcription	Nu	n.d.	n.d.
	Mm.86671	DNA binding	Nu	n.d.	n.d.
	Mm.373242	Transcription	Nu	n.d.	n.d.
Nuclear integrity	Mm.437189	Perinuclear protein	Nu	n.d.	n.d.
Sperm structure or motility	Mm.23534	Tektin3	Cy	+	+ (T)
	Mm.23377	Tep22	n.d.	+	+
	Mm.159795	CatSper3	ER	n.d.	n.d.
Fertilization	Mm.333010	Trypsin-like serine protease	GA	+	+ (A)

**Figure 5:**
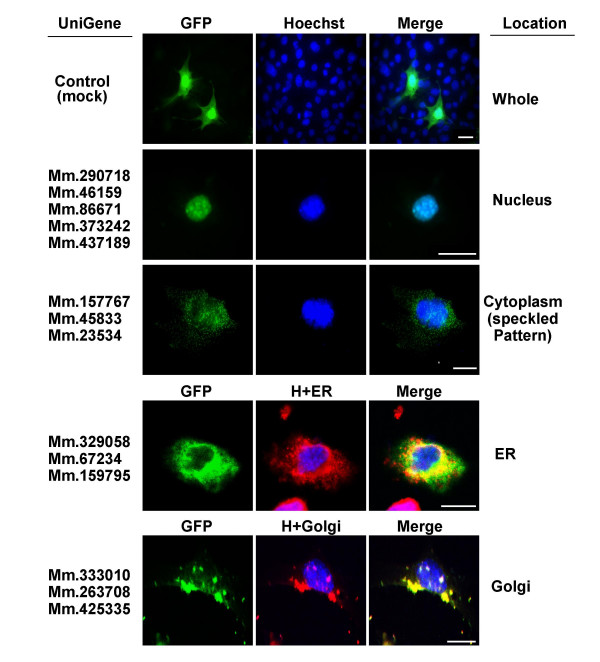
Subcellular localization of the proteins in GC-2 Cells. To determine the subcellular localization of proteins encoded by the 24 genes, GC-2 cells were transfected with each cDNA-GFP in turn. Among these, 14 proteins coded by genes were expressed in GC-2 cells. The figure depicts the localization of these 14 genes. Transfected cells were stained with Hoechst 33258 dye (blue), endoplasmic reticulum marker antibody (antibody to protein disulfide isomerase, red) and Golgi apparatus marker (NBD C_6_-ceramide, red). H, Hoechst; ER, endoplasmic reticulum. Scale bar = 10 μm.

**Figure 6:**
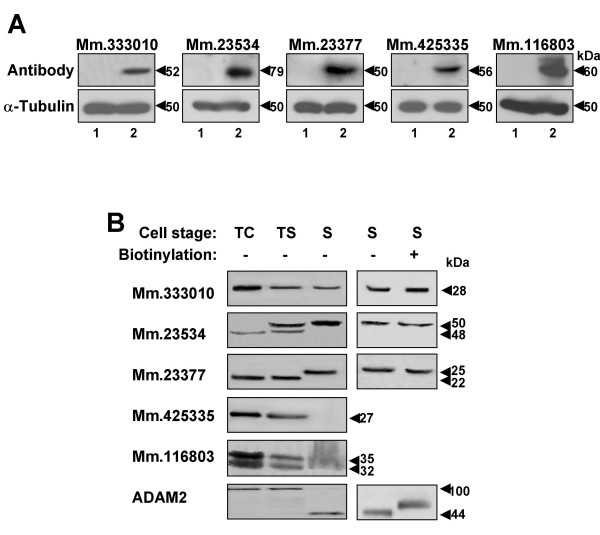
Western blot analysis of the five proteins. **(A) **HEK cells transfected with empty vectors (lane 1) or GFP-tagged cDNA sequences of the five genes (lane 2) were subjected to Western blot analysis, using antibodies to the proteins. The expected molecular weights of the proteins are 53 kDa (Mm.333010), 75 kDa (Mm.23534), 50 kDa (Mm.23377), 52 kDa (Mm.425335), and 57 kDa (Mm.116803). An anti-tubulin antibody (α-Tubulin) was used as a control antibody. **(B) **Protein samples from different stages (testicular spermatogenic cells, testicular sperm, and mature sperm) were blotted with antibodies. Two proteins (Mm.23534 and Mm.23377) displayed alterations in molecular weight. Among the five proteins, three present in sperm were analyzed with regard to their localization. Mature sperm were treated with biotin (+) or left untreated (-), and subjected to Western blot analysis. ADAM2 was used as a control protein. TC, testicular spermatogenic cells; TS, testicular sperm; S, mature sperm; ADAM, a disintegrin and metalloprotease.

**Figure 7:**
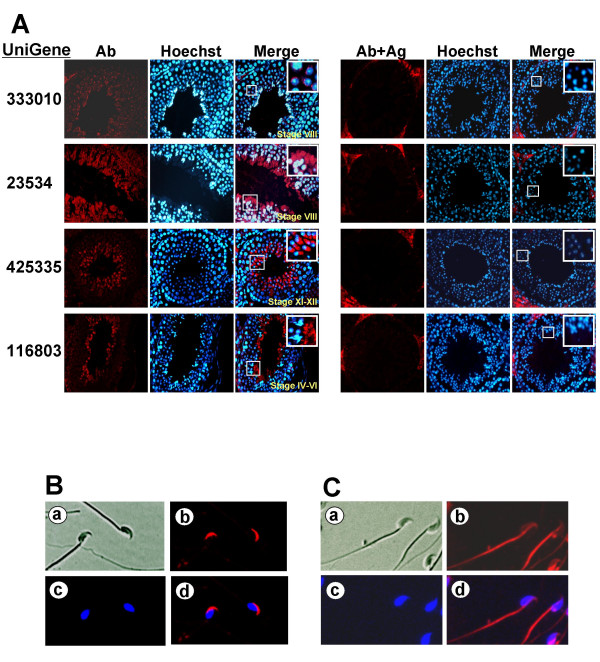
Immunofluorescence analysis of the four proteins. **(A) **Immunofluorescence staining of paraffin sections of adult mouse testis was performed using specific antibodies for the four proteins (shown at the left). Normal serum was used as the negative control. The red color indicates proteins, and blue color represents nucleus staining by Hoechst. The white rectangular boxes are magnified in the insets in merged images. As a further control, the antibodies were pre-treated with the corresponding antigens and used for immunofluorescence staining (shown at the right). This resulted in the disappearance of the signals in spermatogenic cells. Ab, specific antibody to the proteins; Hoechst, Hoechst staining; Merge, merged images between Ab and Hoechst; Ab-Ag, antibodies pre-treated with the corresponding antigens. **(B and C) **Sperm immunofluorescence using antibodies to two proteins. Mature sperm from cauda epididymis were fixed, pemeabilized and incubated with anti-Mm.333010 (B) or anti-Mm.23534 (C) antibody. a, transmission image; b, fluorescence image; c, Hoechst staining; d, merged image between b and c.

Eight genes with *in vitro *protein data (Figs. 5, 6 and 7) which support *in silico *information (Table 1 and Figure 4) are functionally categorized. Abbreviations are as follows: TSC, testicular spermatogenic cells; MS, mature sperm; Nu, nucleus; Cy, cytoplasm; ER, endoplasmic reticulum GA, Golgi apparatus; n.d., not determined; (T), tail; (A), acrosome.

### Subcellular localization of the proteins

To explore protein characteristics *in vitro*, we investigated subcellular localization of the gene products [14]. GFP-tagged full-length gene sequences were transiently transfected into GC-2 cells. GC-2 cells are immortalized germ cells (spermatocytes) of mouse testis [15]. We observed GFP signals from 14 out of the 24 gene products analyzed. By contrast, the GFP signals were not detected in the other 10 genes, suggesting that the expression of these proteins is highly transient, very low in amount or delayed. Figure 5 depicts the subcellular locations identified. Five gene products were found to be localized in the nucleus. Other gene products localized to the endoplasmic reticulum (three genes), Golgi apparatus (three genes), and cytoplasm (three genes). It should be noted that the three genes with cytoplasmic localization displayed a speckled localization pattern (Figure 5). The localization data support results from *in silico *prediction (Mm.290718, Mm.86671, Mm.373242, Mm.437189 and Mm.159795) and further protein analysis (see below).

### Characterization of the proteins in mouse spermatogenic cells and sperm

To further explore the characteristics of proteins encoded by the genes, we generated antibodies against five proteins (Mm.333010, Mm.23534, Mm.23377, Mm.425335 and Mm.116803). Initially, we attempted to generate antibodies to all the proteins, using GST recombinant proteins and synthetic peptides corresponding to the proteins. However, for the other 19 genes, the recombinant proteins were not expressed in a bacterial system or antisera from rabbits immunized with the antigens did not detect corresponding proteins from testis. The antibodies to the five proteins detected distinctive bands in the extracts of human embryonic kidney (HEK) cells transfected with the corresponding cDNA sequences, but not in those of cells transcfected with the empty vector (Figure 6A). We examined the presence and localization of five proteins from total protein extracts of testicular spermatogenic cells, testicular sperm or mature sperm from cauda epididymis by Western blot analysis (Figure 6B). All the antibodies recognized distinct bands in testicular spermatogenic cells. They were of the sizes comparable with those predicted from the cDNA sequences. Two of the proteins (Mm.425335 and Mm.116803) were present in testicular sperm, but not mature sperm. The other three proteins (Mm.333010, Mm.23534, and Mm.23377) were identified in both testicular and mature sperm. Size differences for Mm.23534 and Mm.23377 were evident between testicular spermatogenic cells, testicular sperm and mature sperm, indicating that these proteins undergo changes during sperm development and maturation (see Discussion). To further establish the subcellular localization of the three proteins in mature sperm, cell surface biotin labeling was performed (Figure 6B). If a certain protein is present on the cell surface, labeling with biotin results in a change in electrophoretic mobility. However, we observed no changes in mobility of the three proteins in sperm. ADAM2 was included as a reference protein, since it is known to be processed during sperm maturation and located on the sperm surface. Taken together, our results provide evidence on the localization of these five proteins, both at the developmental stage level (three proteins present at all stages, and the other two restricted to testicular cells and testicular sperm) and cellular level (three proteins present in an intracellular compartment) in sperm.

To confirm and further examine the developmental expression and localization of the five proteins in spermatogenic cells or mature sperm, we performed indirect immunofluorescence analysis in paraffin sections of adult mouse testis with the antibodies. The antibodies to four proteins, corresponding to Mm.333010, Mm.23534, Mm.425335 and Mm.116803, displayed immunoreactivity in spermatocytes and spermatids (Figure 7A). We were unable to observe signals in testis sections incubated with the antibody to the other protein (Mm.23377). In particular, Mm.333010 was observed in acrosomes of round spermatids, while Mm.23534, Mm.425335 and Mm116803 were identified in the cytoplasmic region located to the posterior of elongating spermatids (Figure 7A). Since the Western blot analysis demonstrated the presence of Mm.333010 and Mm.23534 in mature sperm (Figure 6), we performed immunofluorescence on mature sperm. Consistent with the result from testis sections, Mm333010 and Mm.23534 were observed in the acrosomal region (Figure 7B) and flagellum (Figure 7C) of mature sperm, respectively. It is important to note that Mm.333010 is predicted to have a trypsin-like serine protease activity (Figure 4). Table 4 summarizes putative reproductive functions of eight genes with both predicted *in silico *information and supporting *in vitro *protein data obtained in this study (see Discussion).

## Discussion

Previously, several studies have investigated genes expressed at specific stages or in particular cell type during spermatogenesis [4-9]. Although these studies provided inclusive information about the expression profile of a large number of germ-cell genes, comprehensive understanding of spermatogenesis requires further systematic identification and analysis of uncharacterized genes with germ cell-specific expression. UniGene is a large and widely used transcript sequence database containing a large amount of unexplored information about genes. The sequences are divided according to tissue type or developmental stage from the UniGene database, which provides a resource for identifying novel tissue-, cell-, or stage-specific gene transcripts. In the present study, analyzing the mouse spermatocyte UniGene library (Lib.6787), we disclosed that significant proportion (11%) of the spermatocyte genes are testis-specific and about half of the testis-specific genes are unknown or uncharacterized. Previously, a similar approach was applied by our group using the round spermatid UniGene library [11], revealing that 22% (467 of 2124 genes) of genes expressed in round spermatids are testis-specific and functions of 74% of the testis-specific genes are unexplored. In the present investigation, the initial number of uncharacterized, testis-specific genes selected from the earlier version of the spermatocyte library is 134. These 134 genes were narrowed down to 24 authentic genes considered to be abundantly and specifically expressed in the testis by various expression analyses. The other 110 genes were eliminated from consideration because they displayed unreliable coding sequences (53 genes), were undetected or detected with unexpected sizes in the PCR assay (25 genes), not specifically or predominantly expressed in the testis (19 genes) or undetected in the Northern blot analysis (13 genes).

Our data provide extensive information on the 24 genes at the genomic and transcript levels. Genomic analysis disclosed orthologues for 13 mouse genes in the human genome and 11 other were identified as mouse-specific genes. The proportion of mouse genes with a single identifiable ortholog in the human genome is about 80%. The other 20% of mouse genes lack a strict 1:1 relationship, due to differential expansion in at least one of the two genomes [16]. Mostly, those mouse-specific genes were involved in reproduction, olfaction and immunity. Similarly, a global view of human and mouse proteases revealed that the mouse degradome is more complex, and several genes in the mouse genome encode proteases involved in reproductive functions [17]. One such example is the testis-specific or predominant ADAM genes in postmeiotic germ cells [18]. Thus, the 11 mouse-specific genes identified in our study are related to aspects of reproductive physiology. At the transcript level, the Northern blot analysis revealed that four genes are transcribed into products of more than one size. In addition, the analysis demonstrated that transcript sizes from the database are consistent with those determined experimentally for most of the genes. A special feature of genes whose expression is strongly favored in male germ cells is developmentally regulated during meiotic and postmeiotic phases [1,2]. Consistently, the expression patterns of the 24 genes during postnatal testicular development, found in the present study, are indicative of developmental regulation. The pachytene spermatocyte stage is significant during spermatogenesis. It involves genetic recombination, which occurs only in germ cells through cross-over between paired chromosomes and increases RNA and protein synthesis in preparation for the next phase [19]. Transcription of more than half (16 genes) the total genes was found to start from the pachytene spermatocyte stage.

Germ cell-specific and developmentally regulated genes could be directly responsible for the spermatogenesis or fertilization. We also investigated the genes at the protein and cellular levels, providing functional perspectives of the genes. The proteins encoded by 14 out of the 24 genes were analyzed in living GC-2 cells. No expression of the other 10 genes might be due to their peculiar protein natures, such as instability, translational delay and toxicity to the cells. Cellular localization of the 14 genes was divided into nucleus, endoplasmic reticulum, Golgi apparatus, and cytoplasm. To further gain an insight into the characteristics of the proteins, we generated antibodies to five proteins. The Western blot analysis disclosed that two proteins were restricted to testicular spermatogenic cells and testicular sperm, while the others were present at all stages, including testicular spermatogenic cells, testicular sperm, and mature sperm. Results from the immunofluorescence analysis of testis sections and mature sperm corroborate and extend the Western blot data. Taken together, our transfection and immuno-analyses provided new information about 16 genes at the protein and cellular levels (Figs. 5, 6 and 7).

Among the 16 genes with the *in vitro *data, eight genes have *in silico *information congruous with the *in vitro *results (Table 3 and Figure 4). We attempted to categorize these eight genes based on all the *in silico *and *in vitro *data, and relate them to potential functions in reproduction (Table 4). Three (Mm.290718, Mm86671 and Mm.373242) of the gene products are likely to be involved in transcriptional regulation. All of these proteins were found to be localized in the nucleus of GC-2 cells transfected with the corresponding cDNAs. Mm.437189, predicted to be present in a perinuclear region, was targeted to the nucleus of GC-2 cells. This protein might be related to nuclear activity or integrity of spermatogenic cells. According to a recent report, Mm.437189 belongs to the cysteine-rich perinuclear theca family with potential functions in the remodelling of the spermatid nucleus [20]. Three of the genes seem to encode proteins implicated in sperm structure and motility. It should be noted that these three genes have been named and reported previously [21-23]. Nonetheless, we did not eliminate them because we have obtained new information about these proteins in this investigation. Mm.23377, named Tep22, has been suggested to be involved in the biogenesis of the acrosome and the midpiece of the sperm tail [22]. Our Western blot analysis newly revealed that the Mm.23377 protein made as a 22 kDa-protein in testicular cells is changed to a higher molecular weight form between the stages of testicular sperm and mature sperm, suggesting post-translational modification. Consistent with this, the protein contains several putative glycosylation and phosphorylation sites [22]. Mm.159795, identified as CatSper3 [21], was found localized to the endoplasmic reticulum in GC-2 cells in this study. Other CatSper family members, CatSper1 and CatSper2, are known to be specifically expressed in sperm and linked to sperm motility [24-26]. In fact, the expression pattern of the CatSper3 gene and its essential role in sperm motility and male fertility were reported during the preparation of the present paper [27,28]. Mm.23534 has been named Tektin3 which belongs to the TEKTIN family [23,29-31]. Tektin2 and Tektin4 are microtubule- or outer dense fiber-associated proteins in sperm flagella [32,33]. Here, we provide the first information about the Mm23534 protein, Tektin3. This protein was found to be present at the sperm flagella. It should be noted that the molecular size of the Mm23534 protein was increased during spermiogenesis, suggestive of post-translational modification.

Finally, we also obtained original findings on Mm.333010. The protein encoded by Mm.333010 was targeted to the Golgi apparatus in GC-2 cells. The immuno-analysis uncovered that the Mm333010 protein, 28 kDa, is present in both spermatogenic cells and mature sperm. In particular, the protein was located in the acrosomal region of mature sperm. It is important to mention that Mm.333010 is predicted to contain a trypsin-like serine protease domain. The acrosome is a Golgi-derived secretory granule which is formed during spermiogenesis and positioned at the apex of mature sperm [34]. When sperm reach the egg extracellular coat, the zona pellucida (ZP), during fertilization, they bind to it and undergo acrosome reaction, releasing the acrosomal contents at the site of sperm-egg binding. The hydrolytic and proteolytic enzymes comprising the acrosomal contents digest the ZP and, thus, enable sperm to penetrate the ZP. The sperm acrosome contains both unique enzymes and common enzymes present in somatic cells [35]. To date, only a handful of unique enzymes have been identified and enzymes directly responsible for the fertilization process are unknown [36]. Thus, the Mm.333010 protein is a candidate for a type of protease involved in the penetration of the ZP during fertilization.

## Conclusion

Identification of genes with spermatogenic cell-specific expression is crucial to understanding the molecular basis of spermatogenesis and fertilization. Our *in silico *analysis indicates that the proportion of testis-specific genes in the spermatocyte UniGene library is 11% and half of them has been unexplored. We have identified and characterized 24 authentic genes by systematic and integrative approaches, providing insights to their genomic, transcript and protein characteristics. In particular, we predict potential functions of the eight genes, based on our *in vitro *data as well as *in silico *information. Thus, the data provided by this study provide a large resource for further investigations into molecular mechanisms of mammalian male reproduction.

## Methods

### RT-PCR

RT-PCR experiments were performed using cDNA from 8 different tissues (testis, brain, heart, lung, liver, spleen, kidney, and skeletal muscle) of male mouse, as well as cDNA from germ cell-lacking testes of *W/W*^*v *^mutant mice, to determine whether these genes are expressed in somatic cells of testis [37]. To establish specific expression at different stages of spermatogenesis, total RNA obtained from testes of prepubertal and adult male mice (age range 8, 10, 12, 14, 16, 20, 30 and 84 days) was used for reverse transcription. Total RNA extraction was performed using Trizol™ Reagent (MRC) according to the manufacturer's protocol, and cDNA synthesized by random hexamer and oligo(dT) priming with Omniscript reverse transcriptase (Qiagen). Gene-specific primers designed to amplify each region are listed in Table 3. Amplification was performed for 32 cycles of 94°C for 30s, 55, 58 or 60°C for 30s, and 72°C for 1 min. Primers for glyceraldehyde-3-phosphate dehydrogenase (*Gapdh*) as a control were employed as follows: forward, 5'-TGA AGG TCG GAG TCA ACG GAT TTG GT-3' and reverse, 5'-CAT GTG GGC CAT GAG GTC CAC CAC-3'.

### Northern blot analysis

Total RNA was isolated from each tissue using Trizol™ Reagent (Molecular Research Center, Inc.). RNA (10 μg) from testis and liver of male mice was heated at 65°C for 5 min, and separated on a formaldehyde-1.2% agarose gel. The gel was equilibrated for 15 min in distilled water, and twice for 10 min in 1 × SSC solution, and total RNA was transferred to Hybond-XL membrane (Amersham). The probe was derived from products amplified with gene-specific primers (Table 3), and labeled with [α-^32^P]dCTP (Perkin Elmer) by random priming using the Prime-It kit (Stratagene), according to the manufacturer's protocol. Blots were prehybridized for 30-60 min at 68°C in Rapid-hyb buffer (Amersham), and hybridized for 90 min at 68°C in the presence of cDNA probe, following the protocol provided. Blots was washed three times in 2× SSC/0.05% SDS at room temperature for 10 min, and twice in 0.1 × SSC/0.1% SDS at 68°C for 5 min, followed by exposure to Hyperfilm (Amersham) with intensifying screens at -70°C.

### *In silico *analysis

The 24 cDNA sequences were translated into the corresponding peptide sequences, which were analyzed with the diverse computational bioinformatics tools, UCSC [38] and Ensembl [39], to determine intron-exon structures, chromosomal locations, and identify matches with the human chromosomal sequences. Using several computational bioinformatics tools, amino acid sequences deduced from the cDNA sequences of genes were analyzed. PSORT II [40] was applied to predict protein sorting signals, cleavage sites, and intracellular localization. To predict the existence of motifs or domains in translated peptide sequences of genes, CDD [41], Interproscan [42] and PPSearch [43] were employed. SignalP and TMHMM [44] were used to determine the presence of putative signal peptides and transmembrane regions. Goblet [45] was applied to predict gene ontology based on three main classes, specifically, molecular function, biological process and cellular components.

### Cell culture and expression of genes fused to GFP

GC-2 (GC-2spd [ts]) cells were obtained from ATCC (Rockville, MD). Cells were cultured at 5% CO_2 _in Dulbecco's modified Eagles medium (DMEM; Gibco) supplemented with 10% fetal bovine serum at 37°C. The open reading frames of 24 genes were amplified by PCR, and cloned into the pEGFP-N2 vector (BD Clontech). GC-2 cells were transiently transfected with the gene-EGFP construct using Lipofectamine 2000 (Invitrogen), according to the manufacturer's instructions. At 24 h after transfection, cells were fixed with formaldehyde, stained with Hoechst 33342 (Sigma), anti-protein disulfide isomerse antibody (Molecular Probes) and NBD C_6_-ceramide (Molecular Probes), and analyzed for fluorescent signals under the microscope.

### Antibodies

PCR products corresponding to the specific regions of four candidate genes (Mm.333010, Mm.23377, Mm.425335 and Mm.116803) were generated using gene-specific primers designed to add a 5' *BamH*I and a 3' *EcoR*I site. Following digestion, amplified products were ligated into the corresponding restriction sites of pGEX-5X-2 (Pharmacia). The resulting constructs were expressed in *E. coli *BL21. Each GST fusion protein was affinity-purified with glutathione Sepharose 4B, except Mm.333010. Four of the purified fusion proteins were used as antigens for the production of a rabbit polyclonal antibody. The GST-Mm.333010 fusion protein was highly insoluble. The fusion protein was cleaved, loaded on an SDS-PAGE gel, and employed as an antigen to produce a rabbit polyclonal antibody. All antibodies were purified with the corresponding proteins as antigens and an AminoLink Immobilization kit (Pierce). A synthetic peptide (N-terminal ^229^CLRRIEAHLDKANAQLASDR^248 ^of Mm.23534) was used to immunize rabbits. After three immunizations, sera were collected, and polyclonal antibodies purified with the synthetic antigen peptide column using an AminoLink Immobilization kit (Pierce).

### Preparation of protein samples

Testicular cells and sperm were prepared as described [46]. Briefly, the cells are isolated by suspension in 52% isotonic Percoll (Pharmacia) and centrifugation for 10 min (27,000 g, 10°C), and resuspended in Mg^2+^-Hepes buffer. Sperm from the cauda epididymis and vas deferens were directly released into PBS. All samples were directly resuspended in 2× SDS sample buffer, followed by boiling for 5 min, or lysed with a non-ionic detergent (1.0% NP-40) for 1 h on ice in the presence of protease inhibitor cocktails (CALBIOCHEM). Lysed proteins with a non-ionic detergent were centrifuged for 10 min at 12,000 g. The supernatant fractions from the lysate were mixed with 2× SDS sample buffer and boiled for 5 min. Samples were reduced with 5% β-mercaptoethanol. For cell surface biotinylation, samples were incubated with 1 mg/ml sulfo-NHS-LC-biotin (Pierce) in PBS for 30 min at room temperature, washed with PBS containing 20 mM glycine, and resuspended in 2× SDS sample buffer.

### Western blot analysis

Each extract containing approximately 20 μg protein was subjected to 12 or 15% polyacrylamide gel electrophoresis, and transferred onto polyvinylidene difluoride (PVDF) membranes (Bio-Rad). Membranes were blocked in TBS-T (TBS: 50 mM Tris-HCl, pH7.5, 150 mM NaCl and 0.1% Tween-20) containing 5% nonfat dry milk for 1 h at room temperature, and hybridized for 1 h with primary antibodies, followed by three washes for 10 min with TBS-T. Bound IgG was detected for 1 h with alkaline phosphatase-conjugated secondary antibodies (Jackson ImmunoResearch Laboratories). After further washing, alkaline phosphatase activity was detected with NBT/BCIP (Promega Biotech). All primary antibodies were used at 2 μg/ml dilution.

### Immunofluorescence

Paraffin sections of mouse testis (Novagen) were deparaffinized using xylene, rehydrated through a graded series of 100%, 95%, 80%, and 70% ethanol, and incubated in 3% hydrogen peroxide in methanol to quench endogenous peroxidase activity. Tissue sections were heated in a microwave oven for 15 min in 10 mM citrate solution (pH 6) for antigen retrieval. The antibodies for control were pre-treated with the corresponding antigens at 4°C for 2 hrs. After blocking with 5% normal goat serum for 30 min, sections were incubated with the primary antibodies (1:1000 dilution) at room temperature for 1 h, washed three times for 5 min with PBS, and incubated with the Rhodamine Red™-X goat anti-rabbit IgG (1:1000 in PBS; Molecular Probes). Sections were stained with Hoechst 33342 dye (Sigma), and observed for fluorescent signals under the microscope. Mouse cauda epididymal sperm were fixed on slide glass in 4% paraformaldehyde and permeabilized with 0.1% Triton X-100 in PBS at room temperature for 15 min. After extensive washing with PBS, sperm were incubated with normal goat serum at room temperature for 30 min and then exposed to the primary antibody (1:1000) in PBS at room temperature for 1 h. Sperm were washed three times in PBS and incubated at room temperature with Rhodamine Red™-X goat anti-rabbit IgG (1:1000 in PBS; Molecular Probes) for 30 min. After washing three times in PBS, sperm fluorescence was observed under a microscope (DMLB; Leica Microsystems).

## Authors' contributions

EC, JL and JO performed classification and selection of genes in the UniGene library. EC, IP, CH and CY carried out the Northern blot and RT-PCR analysis. EC performed the protein analysis. BNC, DHK, EME and CC conceived and directed the project. EC and CC designed the study and drafted the manuscript. All authors read and approved the final manuscript.
